# Early Days of Two-Dimensional Ion Cyclotron Resonance

**DOI:** 10.3390/molecules26113381

**Published:** 2021-06-03

**Authors:** Geoffrey Bodenhausen

**Affiliations:** Laboratoire des Biomolécules, LBM, Département de Chimie, École Normale Supérieure, PSL University, Sorbonne Université, CNRS, 75005 Paris, France; Geoffrey.Bodenhausen@ens.fr

**Keywords:** two-dimensional Fourier-transform (2D FT) spectroscopy, ion cyclotron resonance (ICR), nuclear magnetic resonance, precursor ions, parent ions, daughter ions

## Abstract

This contribution is an attempt to evoke the favorable atmosphere that prevailed in Lausanne around 1986 and provided the backdrop of our invention of two-dimensional ion cyclotron resonance mass spectroscopy (2D ICR-MS). To avoid a self-centered *histoire d’ancien combattant*, we shall try to emphasize the context: the contributions of key players within our nascent research group at UNIL and the established group of Tino Gäumann at EPFL, the role of external speakers, and the open atmosphere that was not yet polluted by bibliometrics, obsessive concern with impact factors, and top–down management of research. We shall also explain why the idea of 2D ICR-MS has been ignored for many years and still has a limited impact: different scientific cultures in the ICR and NMR communities, different concerns with fundamental vs. applied research, different status of theory and numerical simulations, different levels of commitment of instrument manufacturers, not to mention many theoretical problems that appear to be at least as challenging in ICR as in NMR.

## 1. Introduction

From the day of August 1985 when I left the thriving research group headed by Richard Ernst at ETH, after five intense years in Zurich that allowed me to give countless physical chemistry classes as a teaching assistant, write about 20 papers, and complete a sprawling monograph [1], I knew that I had to cut the umbilical cord (*abnabeln* in German) and develop some novel projects of my own. This sounds simple enough, but as many freshly appointed junior professors will recognize, starting afresh is easier said than done. Not surprisingly, I fell short of my objectives. When moving from ETH to the *Université de Lausanne* (UNIL), I carried with me, as entrenched in my mental luggage, not only my passion for two-dimensional Fourier transformations—which I had stumbled upon in Oxford around 1975 and which I had carried with me to San Diego, Massachusetts, Zurich, and Lausanne—but also my recent fascination for multiple-quantum filtration as a means of identifying multiexponential relaxation [2,3] and my dream that the automated interpretation of two-dimensional spectra by pattern recognition would sooner or later be widely recognized [4].

## 2. A Favorable Environment

At the time, my group at UNIL was housed in an ancient eighteenth century building on the *Place du Château*, in the romantic center of the city of Lausanne, only a few hundred steps from a magnificent thirteenth century cathedral, just opposite the *Château St Maire*, the seat of the government of the *Canton de Vaud*. What used to be my office in those days is now occupied by the *Chancelier* of the Canton. There were initially only two NMR spectrometers, both housed in a fairly decrepit building across the square: an aging narrow-bore 360 MHz and a brand-new wide-bore 400 MHz. The former was used mostly by synthetic organic chemists like Hans Dahn, Hugo Wyler, Manfred Schlosser, Pierre Vogel, and their lively groups, all supported by our heroic engineer Martial Rey. The latter spectrometer was shared with André Merbach and Lothar Helm, who were both interested in chemical exchange reactions under high pressure and who did not care much for the homogeneity of the NMR magnet that suffered from swapping probes.

Located about 7 km away from Lausanne’s city center, the *Ecole polytechnique fédérale de Lausanne* (EPFL) had its own chemistry department that was mostly concerned with chemical engineering and physical chemistry, closer to my heart than synthesis. In those days, my appointment at the *Institut de chimie organique* (ICO) of UNIL seemed to be based on a misunderstanding, which was one reason why I occasionally attended talks on physical chemistry at EPFL. The key players at EPFL were Tino Gäumann and Michael Grätzel, the former an established expert of mass spectroscopy, nicely equipped for ion cyclotron resonance (ICR), the latter interested in (though not yet famous for) solar cells. 

It must have been at EPFL that I listened to a talk by Alan Marshall, at that time at Ohio State University, who must have been invited by Tino Gäumann. Alan’s drawings of ion packets exposed to oscillating electric fields, undergoing acceleration on a spiraling trajectory with an increasing radius, reminded me of the trajectories of magnetization in NMR, much like those my DPhil advisor Ray Freeman used to draw with unmatched talent [5]. Moreover, Alan showed that ions could also be decelerated (“cooled down” or “de-excited”) when applying another oscillating electric field with an opposite phase [6]. Perhaps paradoxically, the initial inspiration for 2D ICR came by drawing analogies not so much with two-dimensional exchange spectroscopy or “EXSY” [7,8] but, rather, with z-filtered correlation spectroscopy or “z-COSY” [9], a method that we were developing in 1987, mostly to investigate spin systems with strong second-order scalar couplings [10]. Although this application has no obvious relevance for ICR, it makes use of some aspects of small nutation angles that can be summarized under the term of “linearity” (vide infra). It took a while to realize that 2D ICR was more closely related to EXSY [7,8], the ancestor of two-dimensional Overhauser effect spectroscopy, or NOESY [11,12], both of which have had considerable impact in NMR.

## 3. A Long Gestation

In fact, my old agendas that I compulsively collected reveal that I had been toying with 2D ICR at least two years before moving from Zurich to Lausanne. I had written a first proposal in 1983 in Tallinn in Estonia, on the occasion of an NMR symposium where I was introduced to Endel Lippmaa, who had a keen interest in ICR [13]. My visit to Estonia was part of an extensive tour of the USSR on the invitation of Yuri Ovchinnikov, a trip that was somewhat impeded by the fact that all flights to the USSR were suspended following the downing of a Boeing 747 of Korean Airlines on 30 August 1983, so that I had to fly to Helsinki and take a boat from there. It felt like a spy story from the days of the Cold War. My agenda mentioned a dinner with Lippmaa, Cibeldina, Eichhof, and Schneider on 28 September 1983. I gave a talk on multiple-quantum NMR and coherence transfer pathways at Lippmaa’s institute, and I wrote “discussions ICR with Pikver, draft proposals 2D-ICR” in my agenda on 30 September. At Lippmaa’s request, I wrote a few pages about 2D ICR in my hotel room and ceremoniously handed them over to him, but he never responded. He was, at that time, mostly concerned with determining the mass of the neutrino by comparing the masses of tritium and helium-3 [14], so he may be forgiven that he paid little attention to such a mundane idea as 2D ICR. This was perhaps the first time that I came to realize how a nice idea can be “crowded out” by other, more compelling ideas. Back in Zurich, I could not find anyone with an interest in ICR, although I discussed the idea with Pablo Caravatti, with whom I worked on pulse sequences to invert families of spinning sidebands in magic-angle spinning NMR spectra [15]. Pablo would later join the ICR department at Bruker in Fällanden, shortly after completing his PhD at ETH under Richard Ernst’s supervision in 1986.

## 4. Only a Dynamic Research Atmosphere Can Make Innovation Possible

**Figure 1:**
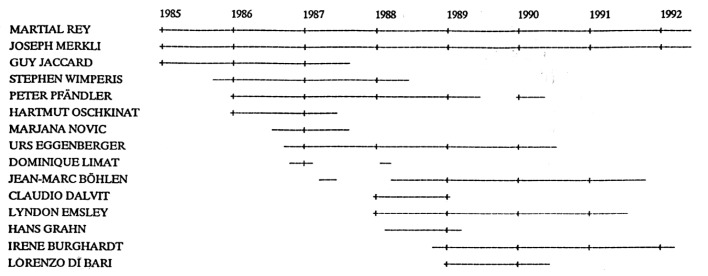
Early coworkers of the Lausanne Spin Department (LSD) at UNIL, only one of whom was reckless enough to embark on the first 2D ICR experiments.

After Alan Marshall’s talk in Lausanne, I tried to convince the members of my tiny group to develop an interest in 2D ICR. My first graduate student at UNIL was Guy Jaccard, who started his PhD under the guidance of my predecessor, Jürgen Lauterwein. Guy was looking forward to completing his thesis, although he was to endure the joys of multiexponential relaxation of ^7^Li before he would be released [16]. The second student was Steve Wimperis, who had spent some time in Southampton and Oxford, where he had worked on broadband Jeener-Broekaert [17] and INEPT sequences [18]. The third student was Peter Pfändler, who had completed his *Diplomarbeit* (M2, in modern parlance) on pattern recognition at ETH [4]. Peter had gone to India for a few months. I wrote a long letter to invite him to join my group in Lausanne. As he would tell me 36 years later, he received my message in a favorable mood, having been mesmerized by the Taj Mahal lit by a full moon. If the Indian postal service had not been so reliable, if Peter had not picked up his *poste restante* messages, if he had decided to return to ETH rather than come to Lausanne, 2D ICR might never have seen the light of day. One may wonder if *Saraswati Devi*, the Hindu Goddess of Knowledge, intervened in our favor. The first post-doctoral fellow to come to our team was Hartmut Oschkinat, later to be joined by Marjana Novic, Claudio Dalvit, and Hans Grahn. The next graduate students, Urs Eggenberger, Jean-Marc Böhlen, Lyndon Emsley, Irene Burghardt, and Lorenzo Di Bari, arrived after the shockwaves of 2D ICR had settled (see Figure 1). 

I was quite unable to tackle the challenges of 2D ICR all by myself, without active support of graduate students or post-docs. The last time I had done any experiments on my own was around 1983 [15]. What else was going on in our tiny group, at a time when I was apparently the only one to believe in 2D ICR? What other ideas threatened to crowd out my pet idea? Steve Wimperis appeared rather reluctant to switch his attention to 2D ICR. In a recent mail of 29 March 2021, Wimperis wrote “the project didn’t appeal to me because of the lack of a clean connection between theory and experiment”. Steve had just succeeded in a remarkable transposition of some peculiar manifestations of multiexponential relaxation, discovered by accident at ETH in triple-quantum filtered correlation spectra (TQC-COSY) of methyl groups in proteins by Norbert Müller in Kurt Wüthrich’s lab at ETH [2,3]. In a clever paper, Steve Wimperis and Guy Jaccard adapted this discovery to isolated spin *S* = 3/2 nuclei such as ^7^Li in viscous isotropic phase [16]. Some 40 years later, I still view this work as one of the pinnacles of my career, although I barely contributed anything. Steve wrote “I always recall that this was the time you got your ‘supervision’ of me spot on. You were going away (to a conference, I think) and quite casually said to me ‘Feel free to finish writing the paper while I’m away.’ I was thrilled: I was still a 1st-year PhD student and yet I was being treated like an equal and trusted with an important task.” Steve was about to discover other curious consequences of cross-correlations (CCCC, as he liked to say), such as relaxation-allowed coherence transfer (RACT) [19,20]. I do not blame Steve for his reluctant attitude towards 2D-ICR. The reader is referred to statistics (*vide infra*) about the relative impact of cross-correlations and 2D ICR. I like to think of Steve’s work as a heroic forerunner of Transverse Relaxation Optimized Spectroscopy (TROSY) [21] that would become famous 10 years later, although Kurt Wüthrich never acknowledged our pioneering work.

Fortunately, Peter Pfändler was willing to give it a try. Since his work on automatic analysis by pattern recognition, based on NMR correlation spectra like *z*-COSY, looked rather promising [22,23], Peter decided that he could afford to divert his attention to ICR for a few months. He was quickly introduced to the secrets of ICR by Raymond Houriet, Jacques Rapin, and Marc-Etienne Walser in Tino Gäumann’s lab. They offered us a first demonstration on an ICR spectrometer at EPFL on 11 April 1986. More experiments were carried out on 25 June, and a whole week was booked on 12–16 January 1987. The data were analyzed by Peter Pfändler, using a rather unreliable computer developed by Bruker (‘workstations’ were not yet widely available in those days), located in our lab in the center of Lausanne. The transfer of large data matrices in incompatible formats by carrying bulky discs required a good dose of fearlessness in those days. 

## 5. The Basic Idea

The 2D ICR method allows one to map correlations between ‘parent’ and ‘daughter’ ions in complex samples without ion isolation. (One may wonder why there are never any sons among the offspring of ions. Whoever invented the expression of ‘daughter ions’ must have been a forerunner of gender equality.) In a static magnetic field, an ion *I^k^* (or any charged particle like an electron or proton) with a mass *m^k^* and a charge *q^k^* moves on a circular trajectory in a plane perpendicular to the field with a cyclotron frequency that is inversely proportional to its mass:

(1) $$\omega^{\kappa }=\frac{q^{\kappa }Β}{m^{\kappa }}$$

A parent ion *I^p^* can be transformed into a daughter ion *I^d^* by fragmentation, protonation, loss of neutral fragments, etc., modifications that can be induced by collisions with neutral molecules or by irradiation with infrared or ultraviolet lasers. In our first experiments, ion fragmentation resulted from collisions with neutral atoms. The transformation of a parent ion *I^p^* into a daughter ion *I^d^* is accompanied by a sudden change in the cyclotron frequency from ω*^p^* to ω*^d^*. Two-dimensional ICR allows one to correlate pairs of frequencies (ω_1_, ω_2_) = (ω*^p^*, ω*^d^*) that are characteristic of parent and daughter ions before and after a change of their mass-to-charge ratios. There are, of course, many similarities between 2D ICR and 2D NMR exchange spectroscopy that allow one to correlate pairs of frequencies (usually chemical shifts) that are characteristic of the environments of nuclei before and after a chemical reaction or cross-relaxation process. 

In its basic form, two-dimensional exchange NMR spectroscopy (2D EXSY) [7,8] uses a sequence comprising three pulses and three intervals:

β − *t*_1_ − β′ − τ*_m_* − β″ − *t*_2_ (2)

Usually, all three pulses have the same nutation angles β = β’ = β’’ = π/2, although this concept has no equivalent in ICR. The key element of the 2D EXSY method is the ‘mixing time’ *τ_m_*, where chemical exchange may occur. (In 2D NOESY [11,12], exchange does not occur through chemical reactions but through cross-relaxation.) In ICR, it is the transformation of parent ions into daughter ions in the interval τ*_m_* that may be monitored. In NMR, it is the partial conversion of the longitudinal component of the magnetization *M_z_^k^* into *M_z_^l^* in the mixing interval τ*_m_* that leads to a cross-peak at the coordinates (ω_1_ = ω*^k^*, ω_2_ = ω*^l^*). Again, there is no equivalent of any longitudinal components in ICR. In NMR, if a fraction *f_kl_* of the magnetization *M_z_^k^* is converted, while a fraction *f_kk_* = (1 − *f_kl_*) remains invariant, this leads to a cross-peak with a relative amplitude *f_kl_*/*f_kk_* compared to the diagonal peak. (This simple rule can be refined to take longitudinal relaxation into account.) Likewise, in ICR, the conversion of a fraction *f_kl_* of parent ions *I^k^* into daughter ions *I^l^* in the mixing interval τ*_m_* leads to a cross-peak at the coordinates (ω_1_ = ω*^k^*, ω_2_ = ω*^l^*), with a cross-peak amplitude proportional to *f_kl_*. To the best of our knowledge, no attempt has been made so far in 2D ICR to normalize cross-peak amplitudes with respect to diagonal peak amplitudes. Line-broadening due to collisions with neutrals and to Coulomb repulsion between ions is usually not taken into account in the analysis of 2D ICR spectra.

In NMR, the three radio-frequency (*rf*) pulses β, β’, and β’’ are characterized by nutation or ‘flip’ angles that are determined by their durations and their amplitudes. The *rf* amplitude is usually chosen to be stronger than the range of offsets, so that the pulses induce the same nutation angles, regardless of offset. The three pulses in Equation (2) usually have the same monochromatic carrier frequency, chosen in the vicinity of the precession frequency of the nuclei under investigation. 

In NMR, 2D EXSY experiments can be described in terms of classical Bloch equations. Each ensemble of identical spins is associated with a classical magnetization vector ***M****^k^* = (*M_x_^k^*, *M_y_^k^*, *M_z_^k^*). Relaxation can be treated in purely phenomenological terms. The transverse components *M_x_^k^* and *M_y_^k^* decay with a time constant *T*_2_, while the longitudinal component *M_z_^k^* returns to its equilibrium at the North pole with a time constant *T*_1_. If relaxation can be neglected, the trajectories of each ***M****^k^* vector can be described on the surface of what is commonly referred to as Bloch’s sphere. The curvature of this sphere is responsible for the nonlinear response of magnetization in NMR. There is no analogy for such nonlinear effects in ICR.

In NMR, the transverse magnetization precesses about the applied magnetic field *B*_0_, in a plane perpendicular to this field, both in the ‘evolution interval’ *t*_1_ and in the ‘detection interval’ *t*_2_. On the other hand, in the ‘mixing time’ τ*_m_*, only longitudinal magnetization that is parallel to the applied magnetic field *B*_0_ needs to be considered. In ICR, one cannot make a distinction between the transverse and longitudinal components. One usually neglects ‘drift’ motions around the radial electric field ‘hill’, orthogonal to the applied magnetic field *B*_0_. 

If 2D EXSY NMR experiments use three pulses with identical flip angles β = β’ = β’’ = π/2, the fate of the magnetization can be readily described. The first pulse β converts *M_z_^k^* into *M_x_^k^* (or *M_y_^k^*, depending on the phase of the pulse), the second pulse β’ has the opposite effect and generates a *t*_1_-dependent longitudinal component *M_z_^k^*(*t*_1_), which, after a partial conversion from *M_z_^k^*(*t*_1_) into *M_z_^l^*(*t*_1_) through exchange or cross-relaxation, is converted by the third pulse β’’ from *M_z_^l^* into *M_x_^l^* (or *M_y_^l^*) that induces a signal at ω_2_ = ω*^l^* in the detection interval *t*_2_. Again, since one cannot distinguish the longitudinal and transverse components in ICR, the analogy seems to break down. 

Although this is not routinely done in NMR, one can modify 2D EXSY by using three pulses with small flip angles β = β’ = β’’ << 90°, say 10°. This can be used to obtain *z*-filtered COSY spectra [9] or to map longitudinal relaxation pathways in systems with scalar-coupled spins [24]. Although 2D EXSY with small flip angles only plays a marginal role in NMR, it offers straightforward analogies with 2D ICR. Indeed, by using only small flip angles in NMR, the excursions of the magnetization vector are limited to the vicinity of the North Pole of Bloch’s sphere. The precession of a magnetization vector leads to a circular trajectory around the North Pole on a plane parallel to the equatorial plane. By focusing attention on this plane, i.e., by neglecting the curvature of Bloch’s sphere, the nonlinear response typical of NMR can be ignored, so that NMR becomes analogous to ICR in this linear regime. Yet, 2D EXSY NMR with small nutation angles cannot be understood without distinguishing the longitudinal and transverse magnetization components, so the similarity between NMR and ICR seems far from obvious.

In both 2D NMR and 2D ICR, the evolution interval *t*_1_ = *n*_1_Δ*t*_1_ is incremented in *N*_1_ steps with *n*_1_ = 0, 1, 2, …, (*N*_1_ − 1), where the increment Δ*t*_1_ determines the spectral width Δν_1_ = 1/Δ*t*_1_ in the *F*_1_ or *ω*_1_ domain after Fourier transformation. The maximum duration *t*_1_^max^ = (*N*_1_ − 1)Δ*t*_1_ determines the digital resolution *δ*ν_1_ = 1/*t*_1_^max^ = Δν_1_/(*N*_1_ − 1) in this domain. Likewise, the signal is observed in the detection interval *t*_2_ by taking *N*_2_ samples at intervals Δ*t*_2_ that determine the spectral width Δν_2_ = 1/Δ*t*_2_ in the *F*_2_ or *ω*_2_ domain. The ‘acquisition time’ *t*_2_*^max^* = (*N*_2_ − 1)Δ*t*_2_ determines the digital resolution *δ*ν_2_ = 1/*t*_2_*^max^* = Δν_2_/(*N*_2_ − 1). In NMR, the spectral widths Δν_1_ = 1/Δ*t*_1_ and Δν_2_ = 1/Δ*t*_2_ are typically on the order of a few kHz, while the linewidths can be on the order of a few Hz, and the data matrices have typical dimensions *N*_1_ × *N*_2_ that are on the order of 128 *×* 1024. In ICR, the spectral widths Δν_1_ = 1/Δ*t*_1_ and Δν_2_ = 1/Δ*t*_2_ may be on the order of several MHz, 1000 times larger than in NMR, while the linewidths can be narrower than in NMR, on the order of a few tens of mHz. For ICR, the data matrices may have typical dimensions *N*_1_ × *N*_2_ on the order of 2 k × 1 M. This represents a challenge for Fourier transformations that was far beyond the resources available in 1987. Comparisons between the resolution in NMR and ICR are only valid if the signal intensity does not vary as a function of time. In NMR, as we have come to realize in recent years, this assumption breaks down for hyperpolarized samples that can be relatively short-lived, while, in ICR, a constant signal intensity can only be maintained if there is a stable supply of freshly ionized ions. In the course of long 2D runs, one may run out of spray solution, or the nano-spray tip may clog.

## 6. Similarities and Fallacies

In ICR, the three pulses with alternating electric fields (with frequencies typically in the range between 10 kHz and about 1 MHz) confer energy to the ions (acceleration or heating) or withdraw energy from these ions (deceleration or cooling) [6,13]. In either case, the energy is determined by the product of the durations and amplitudes of the pulses.

While this analogy has led to the simple (or should we say simplistic?) idea that 2D EXSY could be readily ‘carried over’ from NMR to ICR, it has also given rise to some misunderstandings. In NMR, regardless of whether the description is limited to the polar region of Bloch’s sphere or not, the North Pole acts as an ‘attractor’ [25], since nonvanishing transverse magnetization components that appear as soon as one deviates from the North Pole decay through transverse *T*_2_ relaxation. At any time in NMR experiments, the transverse components can be scrambled (‘purged’ or ‘de-phased’) without affecting the longitudinal magnetization components by applying pulsed field gradients (PFGs), where the homogeneity of the field *B*_0_ is deliberately degraded during a brief interval *τ_PFG_*. Again, there is no analogy in ICR.

In NMR, undesirable transverse components can also be eliminated by phase cycling, i.e., by combining several experiments with the same sequence of pulses and the same intervals but where the phase φ*_i_* of the *i*th pulse in the sequence is incremented from one scan to the next in steps Δφ*_i_* = 2π/*K_i_*, i.e., φ*_i_* = *k_i_*Δφ*_i_* in a series of *k_i_*= 0, 1, 2, …, (*K_i_* − 1) complementary experiments that are otherwise identical [26]. In the special case where *K_i_* = 2 and Δφ*_i_* = π, a phase cycle boils down to a simple phase alternation. This can be used in 2D EXSY, regardless of the flip angle β, to cancel the transverse components in the mixing interval τ*_m_*. As we shall see below, these concepts cannot be transferred from NMR to ICR, for one cannot distinguish the longitudinal and transverse components in ICR.

Two of the most cherished tools of NMR spectroscopists are the refocusing of inhomogeneous effects to form spin echoes, and spin-locking to prevent dephasing. It seems that the discussion about possible ICR analogs that began in 1987 has still not been settled in the ICR community some 34 years later. There are many sources of dephasing of ion clouds, ranging from space charge to background gas collisions that depend on cross-sections. 

## 7. Our First Paper on 2D ICR

On 25 June 1986, Raymond Houriet suggested investigating protonation reactions involving the following species:

A = ^81^Br-pyridine (*m*_A_ = 159 amu, *f*_A_ = 289.7 kHz, *f*_AH_ = 287.8 kHz), and

B = ^79^Br-pyridine (*m*_B_ = 157 amu, *f*_B_ = 293.4 kHz, *f*_BH_ = 291.5 kHz).

These species can be involved in the following reactions:

A^+^ + B → AH^+^ + neutral products (hydrogen transfer from neutral to ion.) (3)

B^+^ + A → AH^+^ + neutral products (proton transfer from ion to neutral.) (4)

**Figure 2:**
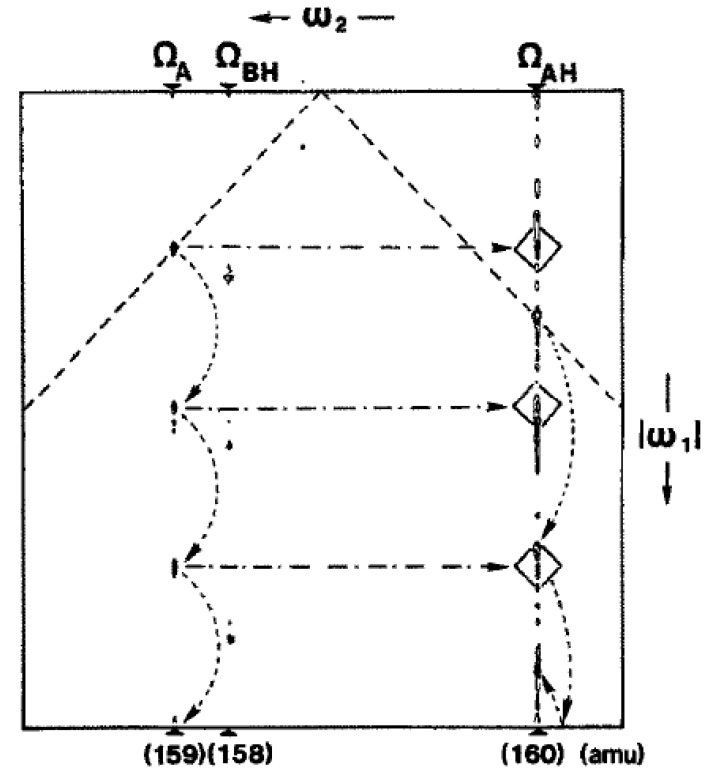
Two-dimensional ICR spectrum resulting from the reactions of Equations (3) and (4). The curved arrows indicate harmonics. The sloping dashed lines represent the diagonal that is partly folded. Reproduced with permission from our first paper on 2D ICR [27].

Our first communication was submitted to Chemical Physics Letters on 2 April 1987, with an accompanying letter to its prestigious editor, A. D. Buckingham in Cambridge, that contained a strange warning: “It might be difficult to find a reviewer for this paper, which is likely to appear obscure for both ICR and NMR people.” It was received on 3 April (air mail did not yet deserve its current name of ‘snail mail’—FedEx and similar private companies cannot guarantee such a quick service, despite their atmospheric fees) and accepted on 30 April [27]. The anonymous referee report recognized some of the weaknesses: “The paper describes a novel application of two-dimensional spectroscopy to ion cyclotron resonance. Because the paper has pioneering character, it should be published despite the fact that the presented two-dimension spectra are not yet convincing in terms of practical applicability and *aesthetic beauty*” (my italics). Indeed, the quality of the first 2D spectra was unbelievably poor (Figure 2). Only with a generous dose of wishful thinking could we see any cross-peaks at all. We boldly (in violation with modern ‘2D NMR ethics’) decided to push the boundaries of interpretation by emphasizing these feeble cross-peaks with hand-drawn boxes. 

Since we used monochromatic pulses, the bandwidth was very limited, so that we could only study a few simple reactions. We therefore developed an alternative scheme, where the frequency was jumped between the second and third pulses of the sequence. There were other problems. In our first paper, we claimed that the second pulse would de-excite the ion packages and that the chemical reactions would involve only ‘cold’ ions, in the sense that they would have a small velocity and small translational kinetic energy, which should not be confused with their internal rovibrational energy. An anonymous expert repeated our experiments and came up with a picture that was diametrically opposed to ours: he/she believed that only ‘hot’ ions that would be excited onto higher orbits would be capable of undergoing reactions. Although I was initially mightily irritated that such an unorthodox theory could have been published in a respectable journal (I believe it was in *J. Chem. Phys.*) without my prior approval, I gradually came to realize that I had no strong arguments in favor of ‘cold’ ions. 

## 8. A Trip to Caltech

On 10 April 1987, just after an ENC meeting in Asilomar, I gave a talk about 2D-ICR at Caltech in Pasadena. I had arranged to be invited by writing to my old friend Manfred Morari, who passed my letter on to Daniel Weitekamp. The latter promptly organized a talk and a meeting with John Baldeschwieler.

On 27 April 1987, after my return to Europe, I sent a copy of our manuscript to John at Caltech, with another unusual comment “You are most welcome to use this paper for your ‘old man’s lecture’ on ICR, even though it has not been accepted yet.” Not shying away from setting up a double-bind situation, I sent a follow-up message by telex (!) to John on 14 May: “Could you please refrain from mentioning our paper (…) in public talks? We are preparing a patent application, and public presentation would be harmful (…) under European law.” 

Furthermore, also on 27 April 1987, I sent a copy of our manuscript to Ernest Laue in Cambridge, in the hope that his skills in maximum entropy methods (MEM) might help us to overcome the limitations of Fourier transformations. Indeed, we realized that, unlike 2D NMR, the modulations as a function of the evolution interval *t*_1_ were far from sinusoidal. We were aware that Fourier transformations were therefore not ideal for the problem at hand, since they gave rise to a series of harmonics. Ernest Laue suggested an elaborate MEM strategy in a letter densely packed with equations, including a promising comment “It should be possible to set up a discrete reconstruction in a better way.” I must admit to my shame that we never tried to implement Ernest’s ideas, which have remained in my files ever since 1987.

## 9. Our Patent Application

Just after submitting our work to CPL, on 4 April 1987, before my trip to California, I sent a copy of our manuscript to H. P. Kellerhals, director of Spectrospin AG in Fällanden, who had a strong interest in ICR. He promptly replied on 14 April and forwarded our manuscript to his patent attorney in Stuttgart on 19 May. (“*Professor Bodenhausen überlässt uns die Idee für eine Patentanmeldung*”— I confirmed my agreement on 5 June.) The attorney was warned that the paper had been accepted and would be published on 10 June. On 3 June, I received a draft (in German) of a patent application comprising 13 pages with six claims. The final application (“*Verfahren zur Aufnahme von ICR-Massenspektren und zur Durchführung des Verfahrens ausgebildetes ICR-Massenspektrometer*”) was filed at the European patent office on 6 June 1987, 4 days before our work was published in CPL. An English translation was filed in the USA. The US Patent 4855593 was awarded 12 years after our filing on 8 August 1989, apparently without review.

A fairly positive examination report by the German patent authorities was issued on 27 May 1988, about a year after our filing. My elaborate reply (in German) was dated 28 July 1989. The German patent DE 37 19 018 C2 was issued on 16 April 1992, 4 years after our filing. I had been worrying, ever since the lecture I gave at Caltech, that my careless disclosure might be exploited by some hypothetical opponent to nullify our patent by invoking my irresponsible violation of ‘absolute novelty’, a concept apparently unknown to US patent authorities but cherished by their German counterparts. To my disappointment, there have never been any opponents, and 34 years later, the question has lost its relevance.

The European patent office (EPO) sent their highly critical comments (also in German) about 7 years after our filing on 5 September 1994. In a nutshell, the EPO examiner was of the opinion that the step from 1D to 2D ICR was trivial. My reply, on the letter heading of the National High Magnetic Field Laboratory (NHMLF) in Tallahassee, was dated 12 October 1994. Since Alan Marshall’s office was only about 10 steps from mine, I could easily consult him. He noted that 13 years had passed between his patent on 1D ICR and our patent application on 2D ICR and 9 years between the 2D EXSY paper [7] and our patent application. Our attorney in Stuttgart wrote a powerful rebuttal in seven densely argued pages. I do not have any records if a European patent was ever awarded.

This was the first patent in my career. For many years, I continued fighting similar cases, convinced that it would be good to show off a few patents to boost grant applications to various agencies, good for the careers of graduate students and post-docs, and, last but not least, good for my own career. After having been awarded no less than 26 patents against increasing odds, I decided that it was no longer worth the effort. I have never earned any significant rewards for my efforts. The fact that I did not expect any further promotions may have played a role.

In the early days, the University of Lausanne did not interfere with matters of intellectual property (IP)—In fact, they did not even have an office to deal with IP. More recently, employers like EPFL, ENS, PSL, CNRS, etc. have come to view IP as a potential gold mine and have developed elaborate rules to prevent any uncontrolled transfer from the public to the private sector. Scientists are no longer allowed to submit their papers until the IP situation has been “cleared” by appropriate offices, which can take several months or even years. While this may be worthwhile for a handful of exceptional inventions, the complexity of the IP rules and the length of the approval procedures has greatly dampened my enthusiasm and inhibited my desire for any further IP adventures. Experiences with French and Swiss authorities have exhausted my energy: a patent on “Heteronuclear Decoupling by Quenching Rotary Resonance in Solid-State Magic Angle Spinning NMR” filed with Piotr Tekely and Markus Weingarth (EP 2 159 589 B1) with no less than four owners (EPFL, ENS, CNRS, and Bruker Biospin) and a patent on a “Method and Device for Performing High Resolution Nuclear Magnetic Resonance with Unknown Spatiotemporal Variations of Magnetic Fields” filed with Philippe Pelupessy (US13/376,741) with the support of the CNRS both demanded a great deal of patience and considerable diplomatic skills. None of our employers or funding agencies have ever been rewarded for their time-consuming and costly attempts to make money on the backs of our ideas.

## 10. Extending the Bandwidth: Our Second Paper on 2D ICR

In our first communication, we used three monochromatic pulses, which severely limited the bandwidth in both dimensions, so that we could only illustrate our idea with rather trivial reactions. Raymond Houriet explained to Peter Pfändler that frequency-modulated “chirp” pulses were routinely used in 1D ICR and that they allowed one to cover bandwidths of several MHz. However, it is far from obvious that one can run an EXSY-type 2D experiment with three chirp pulses. There are lots of sketches in my files that show my skepticism. Fortunately, Houriet and Pfändler went ahead, despite my objections [28]. 

The first broad-band 2D ICR spectra were discussed in internal seminars on 2 March and 28 October 1987. The method was illustrated for the following reactions that may occur when starting with ionized methane CH_4_^+^:

CH_4_^+^ + neutral atoms → CH_3_^+^ + CH_4_^+^ + CH_5_^+^ (5)

and, more interestingly, when starting with CHD_3_ gas:

CHD_2_^+^ + CD_3_^+^ + CHD_3_^+^ + neutral atoms → CHD_2_^+^ + CD_3_^+^ + CHD_3_^+^ + CH_2_D_3_^+^+ CHD_4_^+^ + C_2_H_2_D_3_^+^ + C_2_HD_4_^+^ + C_2_D_5_^+^ (6)

**Figure 3:**
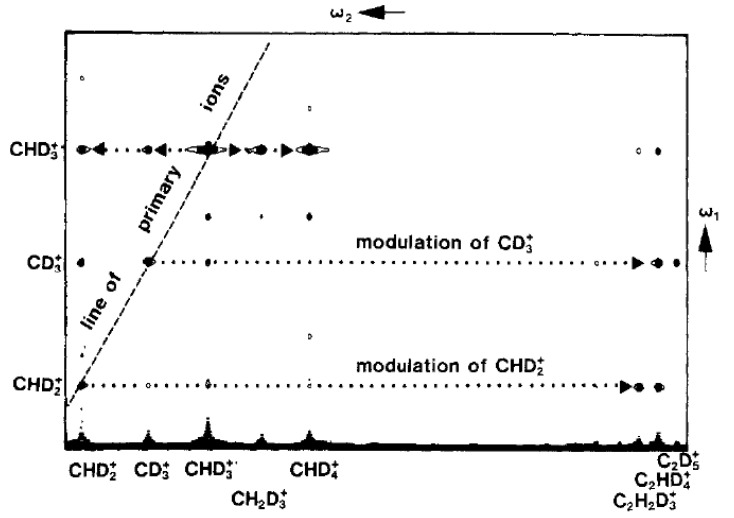
Broad-band 2D ICR spectrum reproduced with permission from our second paper [28].

The resulting 2D spectrum for the latter case is shown in Figure 3.

Our broad-band 2D ICR paper was received by JACS on 17 December 1987 and gave rise to a lively debate. Reviewer 1 wrote: “This paper is definitely appropriate for publication in JACS” but continued with a long list of “minor” revisions, requesting us to explain the presence of axial peaks, discuss harmonics, and cite the extensive history of double resonance in ICR. Let me reproduce the most elaborate comment in full: “It seems to me that the ‘hot’ A^+^ ions raised to higher orbits [by the second pulse], even if they lose their phase coherence during *t_m_*, will still re-acquire phase coherence when excited [by the third pulse], and will therefore be observed along the diagonal of the 2D plot, provided that the excited A^+^ ions are not actually ejected from the cell? The authors might discuss this point.”

Reviewer 2 was much more critical: “Although the present paper has aspects of ‘cross-technology’ and therefore does not deserve the highest marks for creativity, it is a clever adaptation and was made possible by the appropriate collaboration of NMR and MS groups.” This was followed by a few tough statements: “…the first paper [27] is judged to report the breakthrough and this paper is but a modest extension.” (…) “The authors must defend their proposal in terms of advantages in FT ICR.” (…) “The authors must compare their method to the Hadamard proposal [29] which is another means of encoding parent ions but is based on the mechanism of ion ejection.” Then came again a question that sounded familiar by now: “Why are the ‘hot ions’ that are not decelerated [by the second pulse] impossible to detect in contrast to ‘hot’ ions formed in the now classic FT ICR MS/MS experiment, which are detectable?” Finally, the reviewer raised a tricky question: “FT ICR excitation does change the ion energy and, as a result, the rates of ion-molecule reactions.”

Alan Marshall wrote us a long letter on July 1st that explained our experiments better than we had done ourselves, though he was still not convinced that only ‘cold’ ions participated in the reaction. To my perplexity, he wrote “I forgot that ion-molecule collision mechanism switches from Langevin to hard-sphere as the ion orbit increases.” Furthermore, Alan argued that the number of daughter ions produced from a parent ion should depend on the velocity of the parent ion (which increases with the orbital radius). He concluded “that relationship is what generates the parent–daughter relationship in the 2D experiments—the time domain signal of the daughter ions produced by the third pulse is modulated by the sinusoidal variation of the radius of the parent ions produced by the first two pulse.” I quote this letter verbatim 34 years later, because Alan’s interpretation was diametrically opposite to ours. In my reply of August 18th, I argued that parent ions that would be excited onto higher orbits by the second pulse would ‘somehow’ lose their phase coherence. The argument seemed relevant, since daughter ions that appear at arbitrary times “would be scattered all over the cell with arbitrary velocities and phases”. I must admit that, 34 years after exchanging these letters, I am still not quite convinced that my views were correct. 

One minor point: Alan wrote in the same letter “Chirp excitation will be especially unsuitable for this purpose [of covering a large frequency range] because the effect of the chirp is to excite ions well beyond their final orbit before the end of the chirp.” We would soon have the satisfaction to prove that, despite Alan’s warning, chirp pulses are quite suitable!

**Figure 4:**
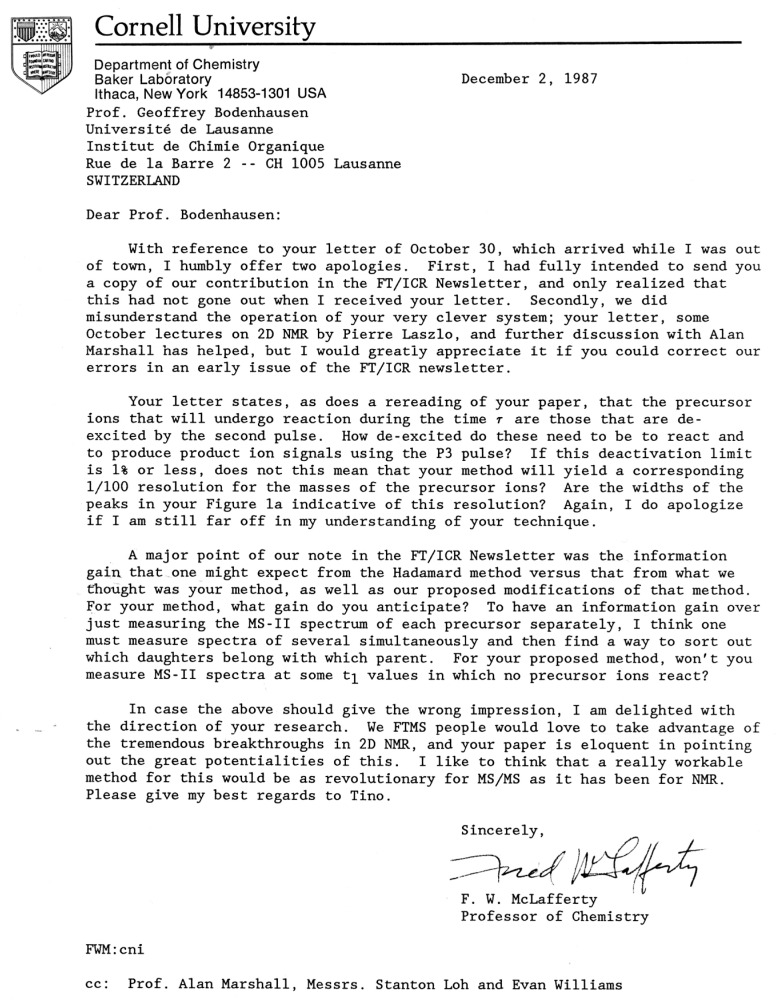
Letter from Fred McLafferty.

In an elegant letter dated 2 December 1987, Fred W. McLafferty of Cornell University humbly offered apologies (this letter, see Figure 4, greatly intimidated me, because I had been taught about “McLafferty *Umlagerungen*” as an undergraduate at ETH): “we did misunderstand the operation of your very clever system” and then proceeded to ask a key question: “[you] state that the precursor ions that will undergo reaction during the time *t* are those that are de-excited by the second pulse. *How de-excited do these need to be to react* and produce product ion signals using the [third] pulse?” (my italics) In other words, how cold must these ions be? That is indeed the crucial question.

In my reply to McLafferty of 14 April, I suggested that there should be a (possibly fuzzy) upper ceiling of the energy of the parent ions and that the lower this ceiling, the weaker the harmonics should be. To the best of my knowledge, this claim has never been put to the test.

Alan Marshall wrote to Tino Gäumann on 27 December 1987, after receiving a preprint of our second paper [28]. He drew attention to prior work on double resonance in ICR, now seemed to accept our view that only ‘cold’ ions contribute to the cross-peaks, worried about the resolution of closely spaced *m*/*z* values, and suggested that ‘hot’ parent ions, even after losing their phase coherence in the *t_m_* interval, may nevertheless acquire a renewed phase coherence after the third pulse, provided that they are not ejected from the cell.

**Figure 5:**
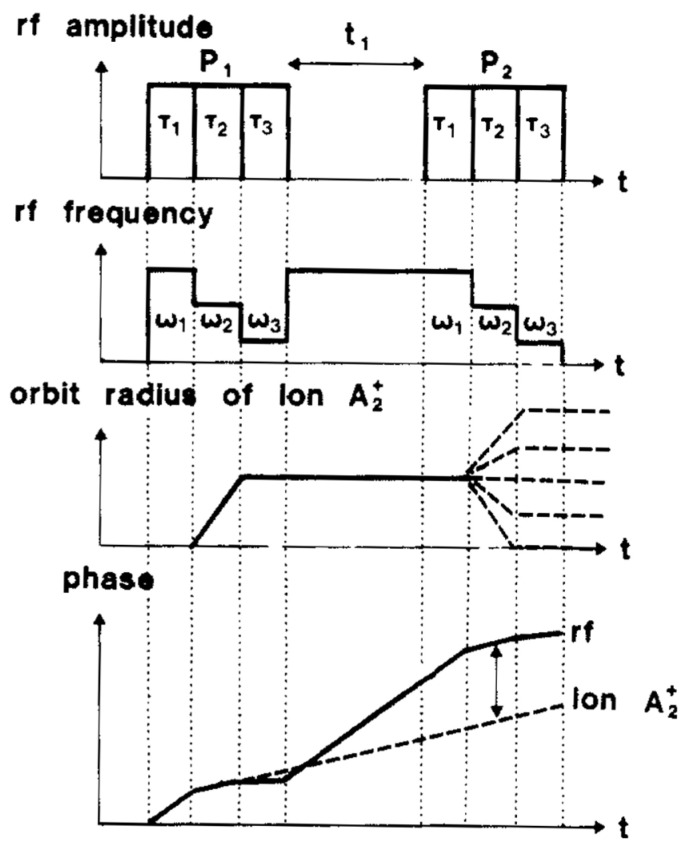
Phase–time diagrams and orbit radii resulting from two consecutive chirp pulses, reproduced with permission from reference [28].

Meanwhile, we struggled with the very concept of chirped pulses in the context of 2D spectroscopy. Figure 5 shows the effects of two consecutive chirp pulses, which actually consisted of a cascade of monochromatic pulses with decreasing frequencies. If an arbitrary ion A^+^ is only affected by the second pulse in each of the two cascades (assuming that the first and third pulses are too far off-resonance to have any effect), the phase–time diagrams and the orbit radii are quite unambiguous.

While the bandwidth problem could thus be overcome with chirp pulses, other problems remained formidable. In contrast to the classical Bloch equations that describe the magnetization vectors in NMR, which invariably return to the North Pole of the Bloch sphere, the ions in ICR experiments have no reason to return to any particular location in the ICR cell. There is no attractor [25], as we would explain much later in 2016. 

**Figure 6:**
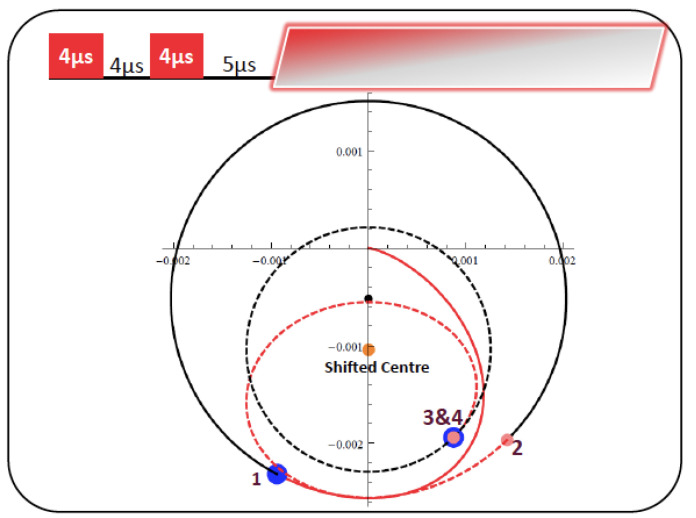
Simulated trajectories that can be off-center not only after the second pulse but even after the first pulse. Unpublished work by Akansha Sehgal, based on parameters similar to those used in her published work [25].

Indeed, extensive (unpublished) numerical simulations by Akansha Sehgal showed that the circular trajectories can be off-center not only after the second pulse but even after the first pulse (Figure 6). We shall leave it to future generations to ponder about the implications.

Furthermore, we realized that the spiraling trajectories are not limited to a plane perpendicular to the static field and that the ions may undergo oscillating motions parallel to the magnetic field. We never found a way to gain control over the third dimension, nor could we agree if this had any relevance to the success or failure of 2D ICR experiments. Attempts to make an ambitious stride from 2D to pseudo-3D ICR in the manner of ‘accordion’ spectroscopy backfired painfully.

A key issue why 2D ICR was not widely understood in 1987 is that fragmentation at a high orbital radius is always a problem in ICR, as daughter ions are ‘born’ with a large moment and are thus generally difficult to detect, as they are likely to be rejected. Thus, modern fragmentation techniques like IRMPD or UVPD, where the ions are fragmented near the axis of the cell, are more suitable for 2D ICR. 

## 11. A Complete Failure in Terms of Bibliometrics!

In retrospect, it seems remarkable that we gave up developing 2D ICR after only two papers on the subject [27,28], until we attempted to make a come-back some 26 years later [30,31,32]. Although I am hardly a fan of bibliometrics and forcefully expressed my fierce opposition to any ‘metrics’, it seems instructive to track the number of citations that mention our two pioneering papers.

**Figure 7:**
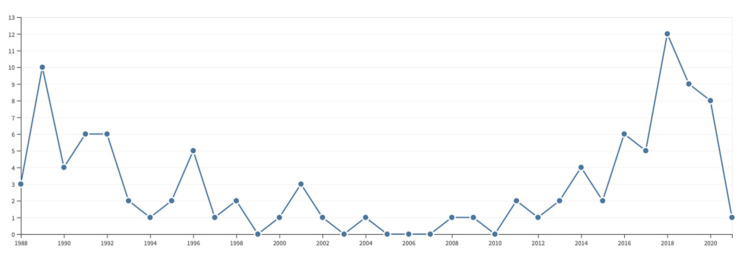
Number of citations aggregated for the two initial papers on 2D ICR [27,28] between 1988 and 2021. The resurgence in 2018 to a modest peak of 12 citations can be ascribed to the papers by Delsuc and Rolando et al. [30,31,32]. Source: Web of Science.

**Figure 8:**
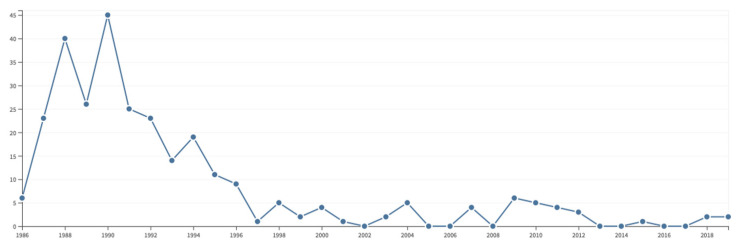
Number of citations aggregated for the 8 papers on the automated analysis of 2D NMR spectra by Pfändler et al. (excluding 2D ICR and pure spectroscopic NMR methods) between 1988 and 2021. The ‘top’ paper, resulting from Pfändler’s undergraduate work at ETH [4], has been cited 79 times. Source: Web of Science.

By today’s standards, a record such as shown in Figure 7 is widely considered as a hallmark of failure! (In 2021, a friendly anonymous reviewer wrote “Just because work isn’t recognized or popular doesn’t make the science a failure. In this case, in particular, it shows work that was *revolutionarily ahead of its time*. As well as far ahead of the computational capabilities of the time” [my italics.]) Although we mercifully did not count any citations in those early days, all of us were (and still are today) obviously concerned with recognition: we hoped to contribute to future breakthroughs or at least to leave a trace of our efforts. It is interesting to see that Peter Pfändler’s main effort on the automated analysis of 2D NMR spectra scored a bit better, with a peak of 45 citations after 2 years, although our hope that pattern recognition would become popular in NMR spectroscopy turned out to be elusive (Figure 8).

**Figure 9:**
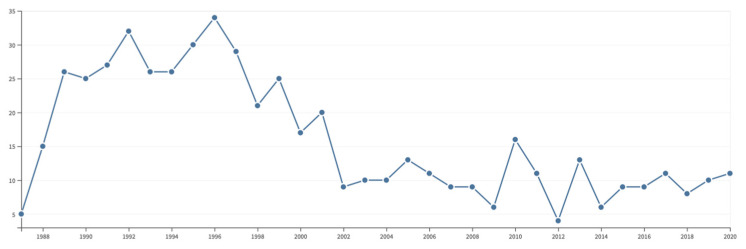
Number of citations aggregated for the 5 papers on multiexponential relaxation by Wimperis et al. (excluding his broadband INEPT and Jeener-Broekaert sequences) between 1988 and 2021. The ‘top’ paper on ^7^Li [16] has been cited 310 times. Source: Web of Science.

Steve Wimperis’ focus on the curious consequences of cross-correlated relaxation effects achieved a somewhat higher integrated score, with a peak of 35 citations after 9 years (Figure 9).

## 12. Obstacles

Why did we not follow up on 2D ICR after our first two pioneering papers? There are at least five factors that contributed to our failure to persist: (i) insufficient understanding of the underlying physics, (ii) lack of motivated students and staff, (iii) lack of interest in the community of mass spectrometry, (iv) lack of interest of instrument companies, and, last but not least, (v) the trap of administrative duties. The following account is, by necessity, rather personal, but I will try to emphasize some obstacles that are likely to be encountered anywhere else, with other people, in other labs, and on other subjects.

## 13. Insufficient Understanding

Despite considerable efforts by Akansha Sehgal, and despite our humble offerings to Saraswati Devi, we never succeeded in providing truly enlightening simulations of ion trajectories in the course of 2D ICR pulse sequences. We neglected ion–ion Coulomb repulsions, although Nikolaev and coworkers [33] have shown how such effects lead to a dispersion of ion clouds and modify both the phases and radii of individual ions. We also neglected axial oscillations along the magnetic field and assumed that the magnetic field is homogeneous across the ICR cell. Recent work by Delsuc and Rolando et al. [32] has successfully addressed the issue of harmonics by developing suitable alternatives to Fourier transformations. We continue to worry about the consequences of the lack of any attractors and still have doubts about the site of the crime: *where* do the fragmentation reactions occur? Thus, to the best of our knowledge, Mc Lafferty’s question (*how cold and how hot?*) has still not been answered.

## 14. Lack of Motivated Students and Staff

After his work on 2D ICR, Peter Pfändler turned his attention back to pattern recognition in NMR spectra, driven by his early passion for a subject that fascinated both of us, though many scientists in our field looked down on us in silent contempt. None of the other people in my group (Figure 1) were willing to take the risk of embarking on 2D ICR. Not so much because they were not intrigued but simply because they had other ambitions, driven by dreams of success and celebrity in other areas of research. Should I have wielded my authority of PhD advisor and exerted more pressure? Should I have told my students to drop their pet subjects and give priority to ICR? I felt intuitively in those days—as I still do—that despotic interventions are likely to be harmful to the motivation of a research student, his most precious gem, the only driving force that matters. Furthermore, new attractive avenues opened up that would carry us back to NMR. Although frequency-swept “adiabatic” pulses have been known since the early days of NMR, we never thought that such pulses might be useful in modern NMR, let alone in modern EPR. The fact that such pulses made a comeback under the playful name of ‘chirp pulses’, a cute expression that came from radar technology, gave them a modern and exciting flavor. In a flurry of hyperactivity, our laboratory developed chirp pulses for the inversion of magnetization [34], chirp pulses to generate spin echoes [35,36], chirp pulses to excite double-quantum coherences [37], and chirp pulses for broadband decoupling [38,39]. With such a full plate, who would be willing to invest his or her precious time in the lost cause of 2D-ICR? Should I have stopped all of these projects on the grounds that they did not address any fundamental questions? 

**Figure 10:**
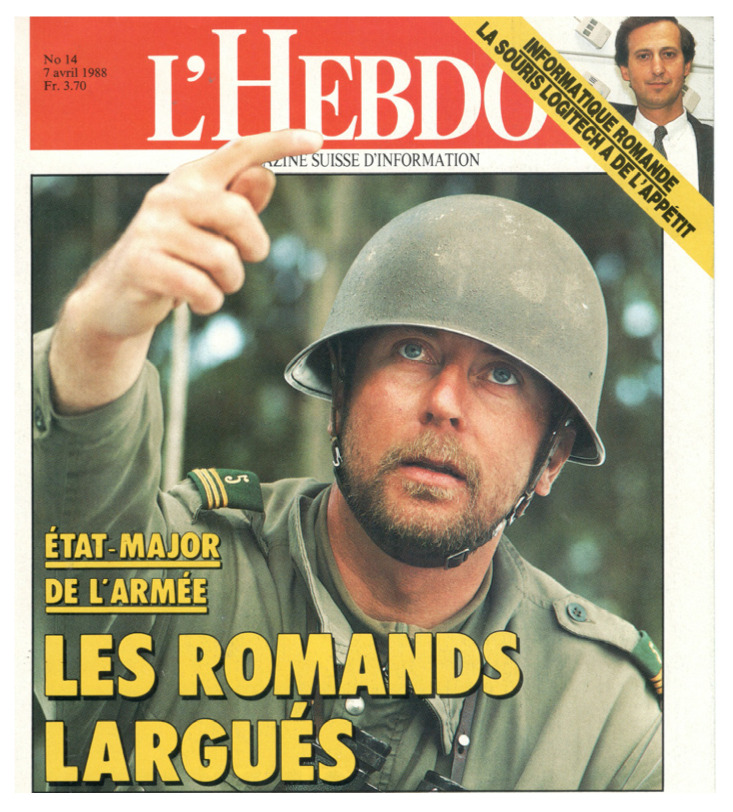
Cover of a Swiss magazine showing Jacques Rapin, one of the inventors of 2D ICR, after joining the Swiss army as a captain.

All PhD supervisors do their best to prepare their students for careers in industry, academia, research, and teaching. It makes sense to encourage initiatives, to involve younger authors by jointly drafting papers and patents, and to encourage their active participation in meetings by posters and lectures. However, some of our former coworkers opted for careers that were quite unexpected. Thus, Jacques Rapin, one of the inventors of our 2D ICR patent, joined the Swiss army (Figure 10). It must be feared that no amount of papers, patents, posters, and lectures could have prepared him for such a challenge. Would Jacques have returned to 2D ICR if his career in the military had not given him full satisfaction? Raymond Houriet rose through the ranks of the higher administration at EPFL. Could he have come back to the laboratory to achieve some unforeseen breakthrough in 2D ICR? Tino Gäumann was approaching his retirement in 1990. He had obtained a key to the seventeenth century baroque church of St Laurent in the center of the city of Lausanne, where he would play on a beautiful organ early in the morning. Had his fingers been less agile on the keyboard, had he experienced some frustration despite his considerable musical skills, would he have turned his attention to 2D ICR? Would Peter Pfändler have done a brilliant post doc in this field? It so happened that his dream to become a chemistry teacher in the gymnasium of his hometown of St Gallen was to become true shortly after he received his PhD degree.

## 15. Lack of Interest in the Academic Community

The academic communities of NMR and ICR turned out to belong to different cultures. In both communities, one ultimately expects to come up with some sensible tools that can provide information about the composition of samples or the structure of matter, be it for analytical chemistry, biochemistry, biology, or medical diagnoses. The NMR community nurtures a remarkably playful attitude towards this grand challenge. People like Freeman, Ernst, Levitt, Morris, Bax, Kay, Frydman, and many others have made their reputation by inventing oodles of seemingly useless pulse sequences. There is a remarkable tolerance to try ideas simply for the sake of the art. The expression *elegant* is quite common in the NMR literature, much more so than down-to-earth expressions like useful or practical. The ICR community, possibly more exposed to the whims of granting agencies, seems to be more focused on ideas of societal or industrial importance. 

## 16. Lack of Interest for Developing Instrumentation

It took about 10 years (1975–1985) for the manufacturers of NMR instruments to develop decent software for 2D Fourier transformations and for programming pulse sequences, shaped pulses, phase shifters, chirp pulses, etc. The manufacturers of ICR instruments took much longer to develop suitable hardware and software, presumably because their customers pushed for improvements in other instrumental aspects of ICR, like efficient coupling with gas chromatography, ionization by electrospray, UV and IR methods. As recently as 2015, we failed to convince Bruker Daltonics to design electronics that would allow computer control of the initial phase of a chirp pulse. (It seems the situation has improved since then.) Consequently, we could not test our ideas on phase cycling. Since the accumulated phase is given by the integral over the time-dependent frequency, chirp pulses that provide a linear frequency sweep in time domain can be programmed in the form of a parabolic time-dependent phase. This has become routine in NMR. In ICR, chirp pulses are generated in a far less sophisticated manner by stepping a frequency source, so that the phase may not be varied in a smooth and continuous manner. The manufacturers of ICR instruments seemed reluctant to overcome the limitations of Fourier transformations and to invest in massive data storage to accommodate large matrices. Presumably, if the scientific community had taken up the idea of 2D ICR with greater enthusiasm, the instrument manufacturers would have responded more keenly, as they did in NMR. Admittedly, although the mass spectrometry community is huge, the ICR community is tiny. Currently, there are only a few dozen dedicated fans worldwide. One day, when the instrumentation and software will have improved, that could possibly change.

## 17. The Trap of Administrative Duties

One of the main obstacles to research is that scientists tend to get trapped in administration. At the time of my appointment in 1985 at the *Institut de chimie organique* (ICO) of the University of Lausanne, my more senior colleagues could never agree on anything. To resolve a stalemate, I was appointed to the job of Director of ICO in 1987, only two years after my arrival, although I was about 10 years younger than my next-youngest colleague Pierre Vogel. With a staff of about 80 personnel, postdocs and graduate students, and a yearly budget for equipment and consumables in the vicinity of a million Swiss francs, I initially felt greatly honored. However, I soon came to realize that, as the youngest man on the block, I had little influence on policy and budget. After being awarded a generous Latsis prize by the Swiss National Science Foundation (SNSF) for NMR and ICR (probably on the initiative of Tino Gäumann), I prepared a speech [40] that made me realize how much my research had suffered from pointless administrative duties. On the occasion of the formal prize ceremony in the *Rathaus* in Bern, I was deeply intimidated by the presence of the President of the Swiss Confederation and a brochette of ambassadors, all of whom seemed to thrive in higher spheres. I resigned from my ICO directorship in a burst of anger. Soon after my resignation, my research group was left out in the cold, exposed to the whims of my successor, who had little appreciation for anything beyond organometallic synthesis. I started looking for a more fertile environment and applied in vain for Tino Gäumann’s succession (EPFL declined my application on the grounds that “NMR belonged to the field of analytical chemistry”, which was at that time considered not to be good enough for EPFL and *une chasse gardée* of UNIL.) I declined job offers at the ENS in Lyon and at the CEA in Saclay and—strongly encouraged by Alan Marshall and by Hans Schneider-Muntau, the chief magnet engineer of the National High Magnetic Field Laboratory in Tallahassee (NHMFL, now simply MagLab)—I moved with my family, lock, stock, and barrel, to the USA in August 1994, thus interrupting (only temporarily, as it would later turn out) our stay in Lausanne. The move to Tallahassee should have opened new perspectives of joint research with Alan’s thriving team, but our ambitious plans to work together on 2D ICR never materialized, in part because, in my capacity of ‘NMR Program Director’, I was expected to start a new group from scratch, while Alan was deeply absorbed by his ICR program. Managerial duties interfered once more. Together with Louis-Claude Brunel, who was responsible for ESR, we agreed to set up a joint structure at NHMFL that we somewhat bombastically called the Center for Interdisciplinary Magnetic Resonance (CIMAR), but we never came around to developing any exciting joint research projects. I came to realize that American bureaucracy was much more time-consuming than its Swiss counterpart, although in the last 30-odd years, the Swiss, French, and European authorities have largely caught up with the USA by creating a flurry of top–down mechanisms to ‘manage’ research, rather than trust individuals to come forward with ideas in a less bureaucratic and far more effective bottom–up approach.

**Figure 11:**
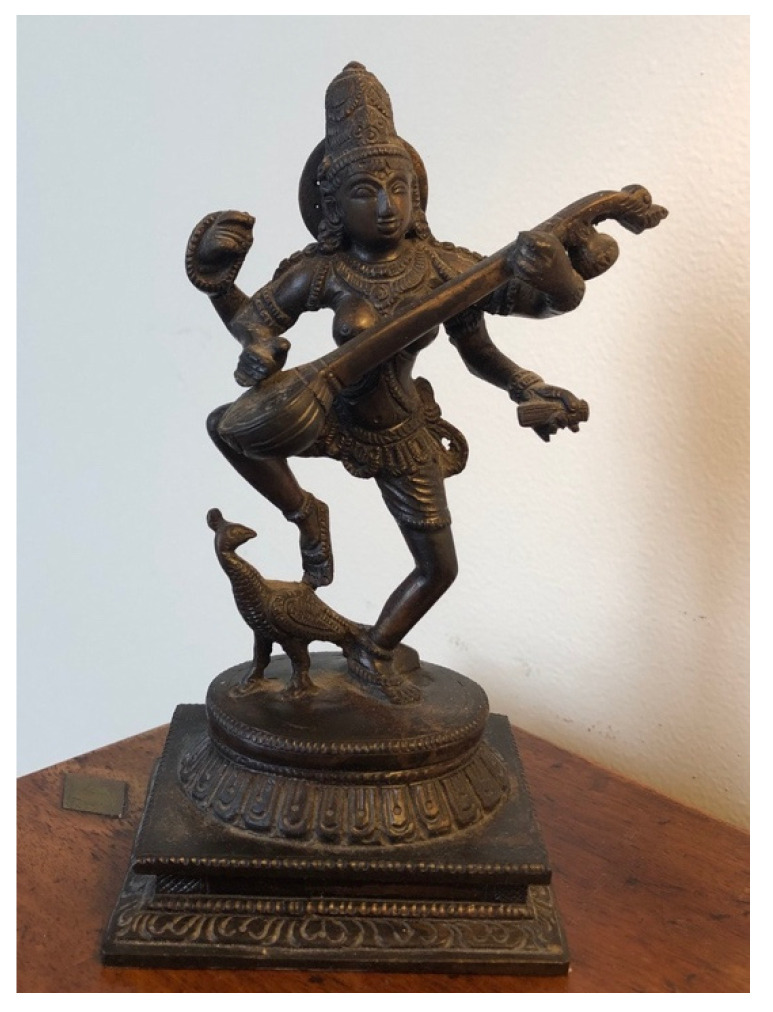
Bronze representation of Saraswati Devi, bought in Bangalore in the 1980s. Anil Kumar wrote in 2021: “You seem to have an unusual statue of Goddess Saraswati, with 4 hands. The artist has the freedom to draw or sculpt his own version of the Goddess. It is a unique piece. Of course, according to our believes, the Goddess bestows wisdom to its worshipers. There is plenty available in your case with so many path-breaking inventions, papers and publications, in addition to training a large number of students and post-docs. You are thus a natural worshiper of Goddess Saraswati.”

Many years later, around 2012, it was at the initiative of Christian Rolando and Marc-André Delsuc that the field of 2D ICR would experience a renaissance. Our joint PhD students Akansha Sehgal and Julien Bouclon bravely traveled back and forth between Paris and Lille, but, despite the benevolence of Saraswati Devi (Figure 11), the inherent difficulty of the subject, instrumental problems, the lack of a common language, and weak management made this joint adventure short-lived, although it may have helped to boost some renewed interest in 2D ICR.

## 18. Conclusions

In summary, the slow start of our 2D ICR ideas in 1983 can be ascribed, in part, to neutrinos in Tallinn, to spinning about the magic angle at ETH, and to the writing of a monograph. The fruitful period in 1986 was brought about by a favorable encounter of a charismatic external speaker, the geographic proximity of two laboratories concerned with ICR and NMR, and the willingness of a graduate student to suspend his thriving research for a few months. Our failure to follow up was due to the multiple factors discussed above, such as competing passions, diverging ambitions, lack of interest in the community of scientists and instrument manufacturers, and a manifold of administrative black holes that absorbed much of our precious energy.

## Data Availability

Not applicable.
